# A Process for Hydrogen Production from the Catalytic Decomposition of Formic Acid over Iridium—Palladium Nanoparticles

**DOI:** 10.3390/ma14123258

**Published:** 2021-06-12

**Authors:** Hamed M. Alshammari, Mohammad Hayal Alotaibi, Obaid F. Aldosari, Abdulellah S. Alsolami, Nuha A. Alotaibi, Yahya A. Alzahrani, Mosaed S. Alhumaimess, Raja L. Alotaibi, Gamal A. El-Hiti

**Affiliations:** 1 Chemistry Department, Faculty of Science, Ha’il University, P.O. Box 2440, Ha’il 81451, Saudi Arabia; h.alshammari@uoh.edu.sa; 2 National Center for Petrochemicals Technology, King Abdulaziz City for Science and Technology (KACST), P.O. Box 6086, Riyadh 11442, Saudi Arabia; aalsolmi@kacst.edu.sa (A.S.A.); nuhaabdullah01@gmail.com (N.A.A.); yalzhrani@kacst.edu.sa (Y.A.A.); raletabi@kacst.edu.sa (R.L.A.); 3 Department of Chemistry, College of Science, Majmaah University, P.O. Box 66, Majmaah 11952, Saudi Arabia; 4 Chemistry Department, College of Science, Jouf University, P.O. Box 2014, Sakaka 72351, Saudi Arabia; mosaed@ju.edu.sa; 5 Cornea Research Chair, Department of Optometry, College of Applied Medical Sciences, King Saud University, P.O. Box 10219, Riyadh 11433, Saudi Arabia

**Keywords:** formic acid decomposition, hydrogen production, renewable energy, green chemistry, catalysis, iridium, palladium

## Abstract

The present study investigates a process for the selective production of hydrogen from the catalytic decomposition of formic acid in the presence of iridium and iridium–palladium nanoparticles under various conditions. It was found that a loading of 1 wt.% of 2% palladium in the presence of 1% iridium over activated charcoal led to a 43% conversion of formic acid to hydrogen at room temperature after 4 h. Increasing the temperature to 60 °C led to further decomposition and an improvement in conversion yield to 63%. Dilution of formic acid from 0.5 to 0.2 M improved the decomposition, reaching conversion to 81%. The reported process could potentially be used in commercial applications.

## 1. Introduction

Fossil fuels are non-renewable energy sources, as these resources will not last forever, and their supply is declining. The expected growth in global energy consumption must be accompanied by the introduction of carbon-neutral energy generation and carrier systems to reduce modern societies’ environmental footprints and overcome the limitations of fossil fuel resources. Renewable biofuels, electricity from nuclear power stations, and solar and wind technology are the most popular contenders for such developments. Although chemical hydrides (CHs) can deliver high gravimetric hydrogen (H_2_) capacities of up to 20 wt.%, the poor reversibility of the processes involved prohibits widespread applications. In this regard, the liquid hydrogen carrier formic acid (FA) has become an attractive choice. FA has a volumetric potential of 53 g H_2_/L, despite containing just 4.4 wt.% of H_2_. This is due to its high density (1.22 g/cm^3^). This equates to a 1.77 kWh/L energy density, which is higher than that of commercial 70 MPa hydrogen pressure tanks (e.g., 1.4 kWh/L for the Toyota Mirai), and could be useful for automobile and smartphone applications. If efficient CO_2_ hydrogenation and selective FA dehydrogenation (FADH) is developed, a carbon-neutral H_2_ storage system would be possible [1]. Therefore, renewable and sustainable energy sources are targets for commercial uses in the near future. Several sustainable energy sources, such as solar and nuclear energy and lithium-ion batteries, have been extensively explored. Recently, the use of hydrogen as an important source of energy for future applications has gained attention. Hydrogen is an environmentally friendly energy source with an energy density of 120 kJ g^−1^ and water as the only by-product of its combustion [2,3]. With a gravimetric energy density of 33.3 kW·h/kg, hydrogen can be converted into energy in an internal combustion engine or fuel cells. However, the ‘hydrogen economy’ is unlikely to emerge before there are major technical advancements in hydrogen processing, storage, and distribution systems. The design of a stable and effective hydrogen storage facility, in particular, is a significant challenge [1].

Hydrogen can be obtained cleanly from the decomposition of formic acid (FA) [4,5,6]. FA is a commodity chemical, found in nature in the venom of ants, and can be obtained as a by-product from bio-refinery processes. Implementing a new strategy that involves the low-temperature synthesis of FA from biomass could enable the use of FA in the industrial-scale production of fuel [7]. However, various limitations hinder the use of hydrogen as an energy source, such as the safe storage and limited capacity of hydrogen and its transportation as an energy carrier [8]. Therefore, significant efforts have been made to overcome such limitations. The most common approaches for hydrogen storage involve the use of sorbent materials [9], metals, and chemical hydrides [10,11].

**Figure 1:**
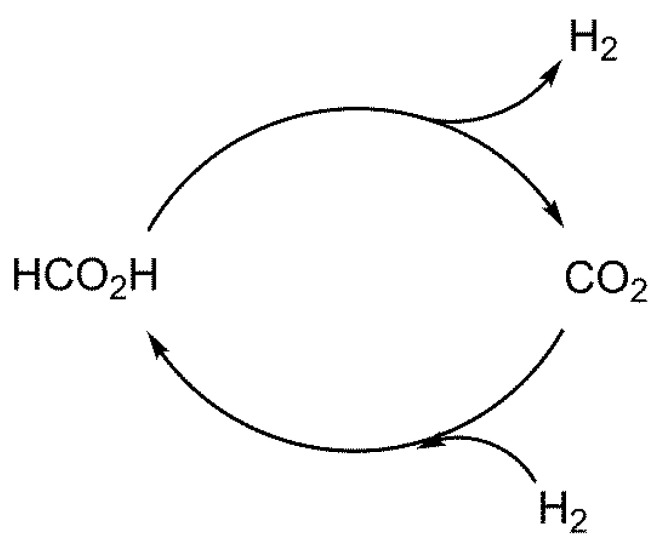
Hydrogen storage cycle.

The use of FA as an effective hydrogen generator for fuel cells is highly important and well documented [12,13,14,15,16,17,18]. FA can be regenerated from carbon dioxide (CO_2_), which is a by-product of its decomposition, via hydrogenation (Figure 1) [19,20,21].

The decomposition of FA involves either the dehydrogenation process, which produces H_2_ and CO_2_ (Equation (1)), or the dehydration process, which produces carbon monoxide (CO) and water (Equation (2)) [22].

(1) $${\mathrm{HCO}}_{2}H\;\left(l\right)\to {\;H}_{2}\;\left(g\right)+{\;\mathrm{CO}}_{2}\;\left(g\right)\;\;\Delta G_{298K}=-35.0{\;\mathrm{KJ}/\mathrm{mol}}^{-1}.$$

Equation (1). Decomposition of FA to H_2_ and CO_2_.

(2) $${\mathrm{HCO}}_{2}H\;\left(l\right)\to {\;H}_{2}O\;\left(l\right)+\;\mathrm{CO}\;\left(g\right)\;\;\Delta G_{298K}=-14.9{\;\mathrm{KJ}/\mathrm{mol}}^{-1}.$$

Equation (2). Decomposition of FA to CO and H_2_O.

Homogeneous catalysts are not a viable option for FA decomposition, because separating them from the reaction mixture is difficult and requires additive(s), ligands, and organic solvents, which are undesirable for industrial applications. Therefore, heterogeneous catalysts are preferable. Ruthenium (Ru) as a catalyst has been shown to have significant activity in the decomposition of FA in the presence of excess amine [23]. The reaction conditions here were optimized to overcome the high volatility of the amine used [24,25] Furthermore, a few studies have investigated the decomposition of FA over solid catalysts in vapor or liquid media at elevated temperatures [26,27,28,29] The use of commercial palladium (Pd) over activated carbon (C; 5% by weight) as a catalyst led to an excellent selectivity (99.9%) toward hydrogen production [30]. Metal–organic frameworks (MOFs) have been used for gas sorption and storage, owing to their high surface area [31]. Metal nanoparticles (NPs) can be loaded inside the pores of the MOFs and used as solid catalysts [32,33,34,35,36]. Pd nanoparticles supported on carbon have been used as catalysts for the decomposition of FA [37]. The use of gold–palladium (Au–Pd) and silver–palladium (Ag–Pd) nanoparticles drives the reaction selectivity toward the desired pathway at a lower temperature [38]. Furthermore, a Co_0.30_Au_0.35_Pd_0.35_ nanoalloy supported on carbon has been used as a selective catalyst in the decomposition of FA to produce hydrogen, with a high conversion rate (91%) at room temperature [29]. Such a catalyst is cheap, easy to prepare, and stable, with no CO produced. Other catalysts such as Ag–Pd core–shells, Ag–Pd bimetallic NPs, Ag–Pd alloy NPs supported on MOFs (MIL-101), and platinum–copper (Pt–Cu) single-atom alloys have been used successfully in the selective decomposition of FA to hydrogen at mild temperatures [39,40]. Sinjay et al. synthesized ruthenium complexes [(η^6^-arene)Ru(κ^2^-L)]*^n^*^+^ (*n* = 0.1) ([Ru]-1 − [Ru]-9) ligated with pyridine-based ligands and used them to produce hydrogen from formic acid in water. They obtained a very effective and stable catalyst in water, which could be used up to seven times, and they achieved a total turnover frequency (TOF) of 6050 h^−1^ [41,42].

In this paper, we report the successful use of iridium (Ir) and Ir–Pd nanoparticles as catalysts in the decomposition of FA using impregnation and sol-immobilization techniques under various reaction conditions. Various techniques, such as scanning electron microscopy (SEM), energy-dispersive X-ray (EDX) spectroscopy, Fourier-transform infrared (FTIR) spectroscopy, Brunauer–Emmett–Teller (BET) surface area analysis, and inductively coupled plasma (ICP) were used to characterize the catalysts.

## 2. Materials and Methods

### 2.1. Materials

Formic acid (≥98%), iridium (III) chloride, palladium (II) chloride, and hydrochloric acid were purchased from the Sigma-Aldrich Chemical Company (Gillingham, UK). Active carbon was purchased from Alpha Chemicals (Gujarat, India). Ultrapure water was obtained using the Milli-Q^®^ Advantage A10 Water Purification System (Merck, Darmstadt, Germany).

### 2.2. Catalyst Preparation

#### 2.2.1. Impregnation Method

A wet impregnation technique by chemical reduction using sodium borohydride as reducing agent was employed: iridium (III) chloride (1% mole of Ir) and palladium (II) chloride (1% mole of Pd) were added to a mixture of 100 mL of distilled water and 1 g of active carbon in a flask (250 mL) and stirred vigorously. After 1 h, a freshly prepared aqueous solution of NaBH4 (0.2 M) was added and stirred for 30 min. The catalyst was filtered and washed with distilled water (1 L). The sample was dried overnight at 100 °C.

#### 2.2.2. Sol-Immobilization Method

Different ratios of iridium (III) chloride (1%, 2%, and 4% mole of Ir) and palladium (II) chloride (1%, 2%, and 4% mole of Pd) were added to distilled water (100 mL) in a flask (250 mL). A freshly prepared solution of PVA (1 wt.%, Aldrich, Mw = 10,000, 80% hydrolyzed) was added to an aqueous solution of PdCl_2_ and mixed with iridium (III) chloride, stirring for 15 min (PVA/metal 0.65 *w*/*w*). A concentration of 0.1 M of NaBH₄ (Sigma-Aldrich, St. Louis, MO, USA, NaBH₄/(Ir + Pd) (mol/mol = 5)) was freshly prepared and then added, to form a dark-brown Pd(0) solution. Then, the active carbon was added to the mixture and stirred for 1 h. After 30 min, the mixture was acidified with sulfuric acid. The paste-like material obtained was washed with distilled water (1 L) and dried overnight at 100 °C.

### 2.3. Catalytic Decomposition of Formic Acid

The catalyst was added to different concentrations of FA (10 mL of 0.2, 0.5, 1, 2, and 25 M) in a two-necked round-bottom flask (50 mL) equipped with a water condenser in an oil bath. The mixture was stirred at various temperatures (25–80 °C) for different reaction times. Each reaction was repeated three times to obtain reproducible and consistent results.

### 2.4. Product Analysis

An aliquot (200 μL) was withdrawn from the reaction mixture at various reaction times, diluted with ultrapure water (4 mL), transferred to a volumetric flask (50 mL), and topped up with ultrapure water. The sample was analyzed by high-performance liquid chromatography (HPLC, 1260 Infinity, Agilent) using an Agilent Hi-Plex H column (300 mm × 7.7 mm) at 50 °C at a flow rate of 0.5 mL/min.

### 2.5. Gas Analysis

The gases produced from decomposition of FA were collected through the displacement of water in a gas burette system. A GC Agilent 7890A gas chromatograph equipped with a GC packed column in stainless steel tubing (1.83 m length, 1/8 inch, OD, 2 mm ID), a Hayesep Q packed column (mesh size 80/100), and Hayesep (3 ft × 1/8 in × 2.0 mm) was used to analyze the gases. Argon was used as the carrier gas and was connected to a methanation unit fitted with a thermal conductivity detector.

## 3. Results and Discussion

### 3.1. Catalyst Characterization

**Figure 2:**
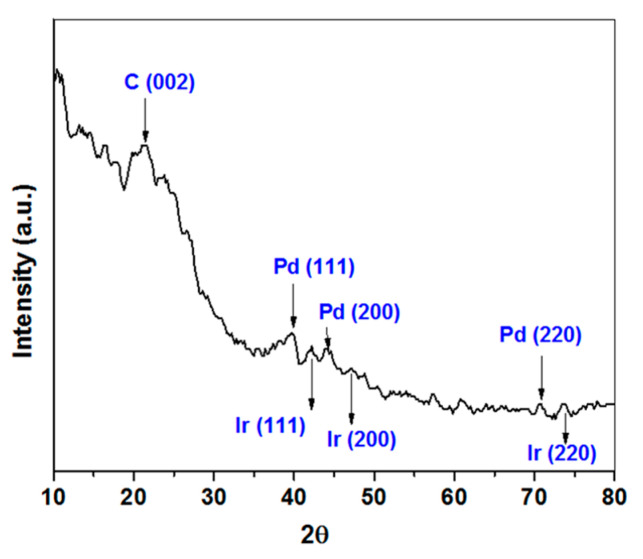
X-ray diffraction patterns of 1% Ir: 2% Pd/C sample.

An Ir:Pd/C 1:2 (1 wt.%) XRD diffraction pattern was observed, as shown in Figure 2. The samples’ diffraction peaks at 22.2° for the prepared catalyst revealed an active carbon structure. The (111), (200), and (220) planes of the face-centered cubic architecture of Pd were assigned to Pd peaks at 40.1°, 45.6°, and 69.9°, respectively (JCPDS card#46–1043). Moreover, the diffraction peaks at 2θ of 41.7°, 47.1°, and 70.1° could be attributed to the (111), (200), and (220) planes of face-centered cubic Ir, respectively.

**Figure 3:**
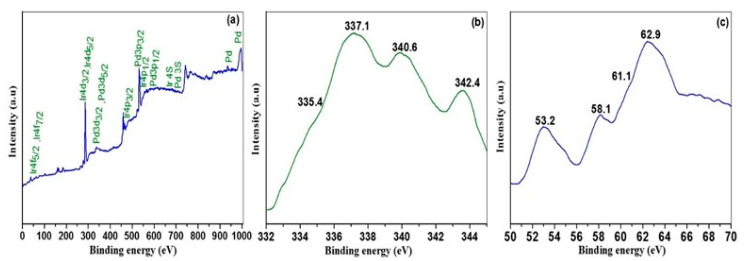
XPS analysis of (**a**) 1% Ir:2% Pd/C, XPS survey spectrum; (**b**) Pd 3d spectrum; and (**c**) Ir 4f spectrum of the same catalyst.

The nature of the surface oxidation state for the 1% Ir:2% Pd/C catalyst was investigated using XPS; the XPS survey spectrum of Ir:Pd/C 1:2 (1 wt.%) is displayed in Figure 3a, while the Pd 3d spectrum and the Ir 4f spectrum of the same catalyst are shown in Figure 3b,c, respectively. The XPS spectrum of Pd 3d is displayed in Figure 3b. Two major doublets were found in the Pd 3d spectrum, suggesting two separate Pd oxidation states: Pd(II) and Pd(0). Furthermore, because Ir metal is readily oxidized to Ir-oxide (IV) in ambient conditions, the XPS spectrum of Ir 4f can be deconvoluted by two sets of curves based on the existence of the oxidized Ir(IV) and metallic Ir. For the Ir:Pd/C (2:1) catalyst, the peaks at 61.1 and 62.9 eV are attributed to the Ir and the peaks at 53.2 and 58.1 eV are attributed to the Ir(IV) oxidized form.

### 3.2. Catalyst Activity in FA Decomposition

**Figure 4:**
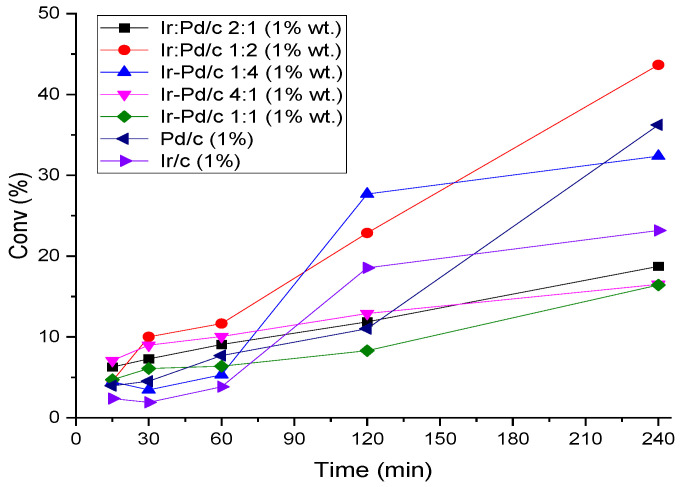
Catalytic decomposition of FA. Reaction conditions: FA (0.5 M), catalyst (substrate:metal molar ratio = 2000:1), 25 °C, 700 rpm, and 240 min.

Two catalysts were prepared with Ir:Pd in a 1:1 ratio over activated charcoal using impregnation and sol-immobilization methods. Both catalysts were used to decompose formic acid (10 mL, 0.5 M, and 5 mmol) at 25 °C, and the reaction mixture was stirred at a speed of 700 rpm for 4 h. Carbon dioxide and hydrogen were the only products detected in the gas phase, with no evident carbon monoxide formation. The results show that the catalyst, which was prepared with the sol-immobilization technique, decomposed the FA more efficiently, by 16.42%, whereas the catalyst prepared with the impregnation method decomposed FA by only 6.6%. A similar observation was previously published in the literature [36]. Therefore, we used the sol-immobilization method for all further experiments. Several Ir and Ir–Pd catalysts over activated charcoal were prepared and tested for the decomposition reaction of FA in the liquid phase. The catalyst was added to FA (10 mL; 0.5 M, 5 mmol) with a substrate:metal molar ratio of 2000:1 at 25 °C, and the reaction mixture was stirred at a speed of 700 rpm for 4 h. Carbon dioxide and hydrogen were the only products detected in the gas phase, with no evident formation of carbon monoxide. It can be seen from the graph in Figure 4 that Pd/C showed a higher decomposition activity than Ir/C; the results were 36% and 23%, respectively, after 240 min. In bimetallic catalysts, better activity was observed. For example, Ir:Pd/C 1:2 (1 wt.%) exhibited the highest activity, with approximately 43% of the FA being converted into H_2_ and CO_2_. No further improvement in conversion was observed when the amount of Ir in the bimetallic catalyst was increased. Pd was present at 335.5 eV (Pd^0^) and 337.1 eV (Pd^2+^), which was different from that of the monometallic catalyst, where both Pd–Cl and PdO were observed. Notably, the Pd(0) binding energy was somewhat higher than what was expected for metallic Pd particles, which may be attributable to Pd^2+^ species formed by a charge transfer with Cl-1 that remained on the surface or the particle size-dependent screening effects of the Pd core–hole that resulted in higher binding energies for smaller particles [43]. Therefore, the Ir:Pd/C 1:2 catalyst was used in further investigations of FA decomposition.

**Figure 5:**
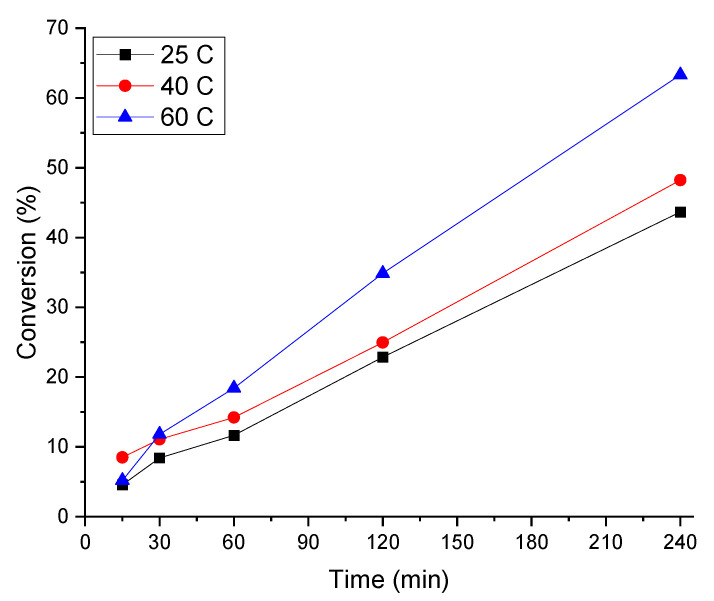
Effect of temperature on FA decomposition over 1%Ir:2%Pd/C. Reaction conditions: FA (0.5 M), catalyst (substrate:metal molar ratio = 2000:1), 700 rpm, and 240 min.

The effect of temperature on FA decomposition was investigated using the Ir:Pd/C 1:2 (1 wt.%) catalyst. The temperature was varied from 25 to 80 °C for 4 h. The results are presented in Figure 5. When the reaction was conducted at 25 °C, the conversion rate of FA to H_2_ and CO_2_ was only 43.6%. Increasing the temperature further to 40 °C led to an improvement in FA decomposition (48.1%). The decomposition of FA was improved further when the temperature was 50 °C, reaching 63.3% after 4 h. We concentrated our research on mild conditions (RT), because operating at moderate reaction conditions is one criterion for portable devices using formic acid in fuel cells.

The activation energy (ΔG), calculated from the slope of the Arrhenius plot, was found to be 4.2 KJ/mol. The TOF and ΔG results, along with the reported values obtained for the decomposition of FA over other catalysts, are shown in Table 1.

**Figure 6:**
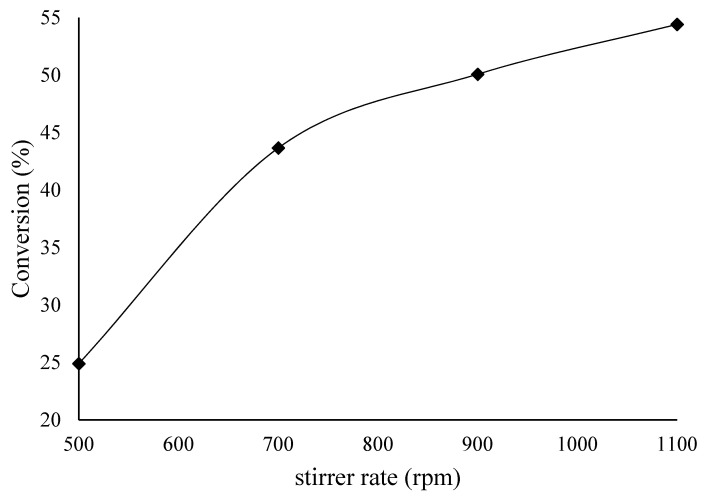
Effect of stirring speed on FA decomposition over 1%Ir:2%Pd/C. Reaction conditions: FA (0.5 M), catalyst (0.1 g; substrate:metal molar ratio = 137:1), and 240 min.

**Figure 7:**
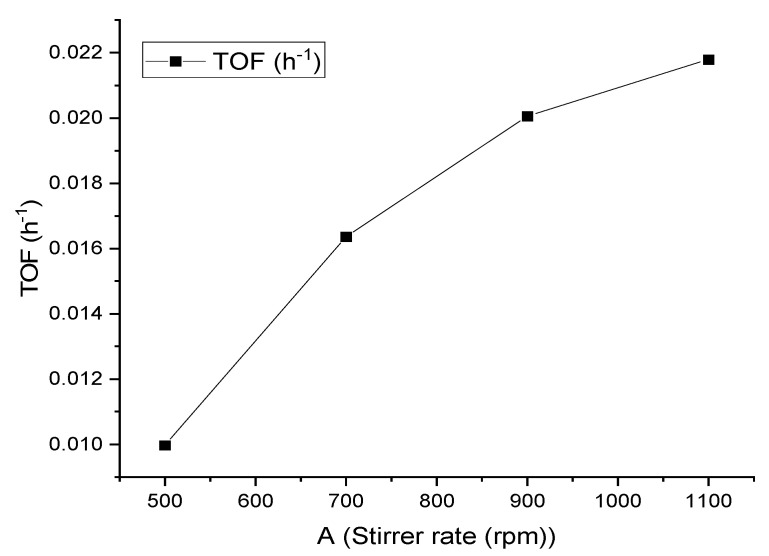
Turnover frequency of FA decomposition over 1%Ir:2%Pd/C. Reaction conditions: FA (0.5 M), catalyst (0.1 g; substrate:metal molar ratio = 137:1), and 240 min.

Next, we investigated the effect of stirring speed on the decomposition of FA. A range of stirring speeds (500–1100 rpm) were tested. These results are shown in Figure 6, and the TOF is presented in Figure 7. As can be seen, the highest conversion was obtained at a maximum stirring speed of 1100 rpm.

**Figure 8:**
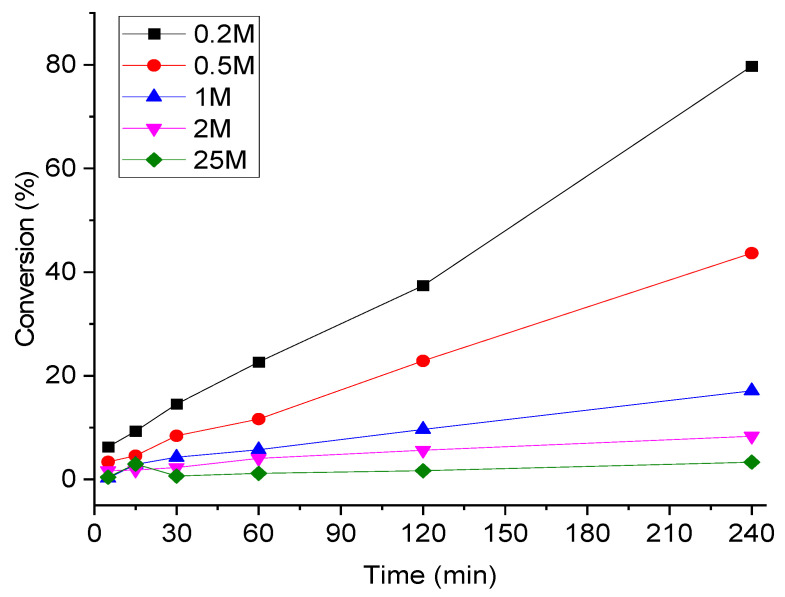
Effect of FA concentration on the decomposition process over 1%Ir:2%Pd/C. Reaction conditions: catalyst (substrate:metal molar ratio = 2000:1), 700 rpm, and 240 min.

In another attempt to optimize the reaction conditions, we investigated the effect of FA concentration on the decomposition process. Various concentrations of FA (0.2, 0.5, 1, 2, and 25 M) were tested, and the results are shown in Figure 8. The results clearly indicate that the highest conversion was achieved when FA was highly diluted (0.2 M).

**Figure 9:**
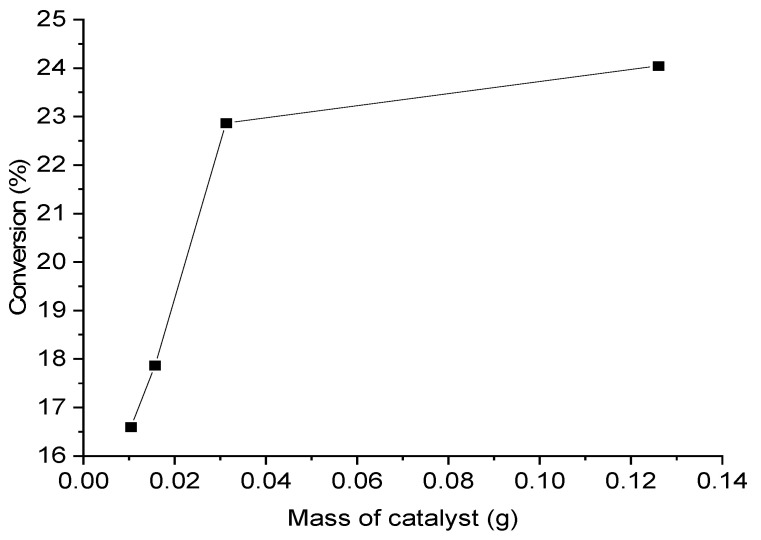
Effect of catalyst mass on conversion, in substrate:metal molar ratios of 500, 2000, 4000, and 6000. Reaction conditions: 25 °C, 0.5 M FA, 700 rpm, and 120 min reaction time.

The experimental data were verified in the absence of external mass transfer limits to calculate intrinsic kinetics (chemical kinetics regime). As a result, the mass transfer limits (external) were tested experimentally. First, the effect of catalyst mass (substrate:metal molar ratio) was studied at 25 °C and 700 rpm, for a reaction time of 120 min. Figure 9 depicts the two reaction regimes. The conversion increased linearly with increasing catalyst mass, i.e., up to 31.2 mg (substrate:metal molar ratio of 2000:1) in the first regime, indicating that the reaction was not mass transport constrained. External diffusion limitations were apparent in the second regime; the conversion did not adopt a linear increase with catalyst mass.

**Figure 10:**
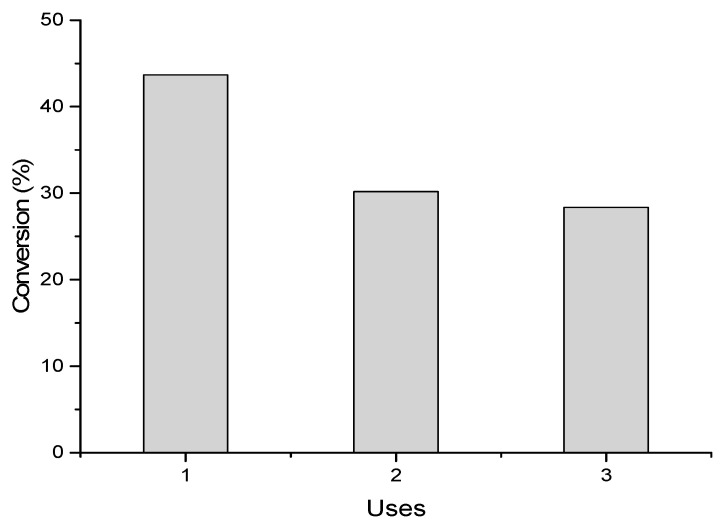
Reusability of 1%Ir:2%Pd/C after three reactions. Reaction conditions: 31 mg of catalyst, 25 °C, 0.5 M FA, 700 rpm, 2 h reaction time.

The catalyst activity after further reuses was studied at 25 °C and 700 rpm, with a concentration of 0.5 M formic acid (substrate:metal molar ratio = 2000:1) and a reaction time of 2 h. The reusability test was conducted by filtering the catalyst at room temperature and atmospheric pressure and using it in a fresh reaction under the same reaction conditions. showed some loss in their activity after the first use, as shown in Figure 10. The catalysts stabilized after the second run. The loss of their activity might be due to increased particle size or agglomeration, the formation of metal species, or the adsorption of formate species [50] For several potential reasons, such as the loss of the active species through reduction, Pd particle sintering, or the active Pd site being covered by coke or adsorbed reactants/products, are reported in the literature, [52,53].

## 4. Conclusions

The decomposition of formic acid was carried out selectively to produce hydrogen and carbon dioxide using 2% Ir:1% Pd (by weight) loaded over activated charcoal at 25 °C for 4 h. The Ir–Pd bimetallic catalysts were stable and showed powerful interactions and high dispersions on the charcoal support. The selective production of hydrogen from the complete catalytic decomposition of FA could be used as an efficient and valuable process for the production of clean fuel.

## Figures and Tables

**Table 1:** Catalytic activities of different types of catalysts for the decomposition of formic acid.

Catalyst (wt.%)	T (°C)	FA (M)	TOF (h^−1^)		ΔG (KJ/mol)	Ref
			Initial	2 h		
2%Ir:1%Pd/C	25	0.50	–	4.12	4.2	This work
Pd_IMP_/CNF	30	0.50	563.2	–	27.5	[38]
Pd_SI_/CNF	30	0.50	979.1	–	26.2	[38]
Pd_SI_/AC	30	0.50	240.5	–	–	[38]
Pd/C	21	1.33	18	15 ^a^	53.7	[44]
Pd/C	30	1.33	48	28 ^a^	–	[45]
Pd/C (citric acid)	25	–	–	64 ^b^	–	[46]
Pd/C	30	1:9 ^c^	–	228.3	–	[47]
Au_41_Pd_59_/C	50	1.0	230		28 ± 2	[48]
Ag@Pd (1:1)	35	–	–	156 ^d^	30	[21]
Ag@Pd (1:1)	50	–	–	252 ^d^	–	[21]
Ag/Pd alloy (1:1)	20	–	–	144 ^d^	–	[21]
Ag_42_Pd_58_	50	1.0	382	–	22 ± 1	[48]
Pd-MnOx/SiO_2_-NH_2_	20	0.265	140	–	61.9	[49]
Pd-MnOx/SiO_2_-NH_2_	50	0.265	1300	–	–	[50]
Ag_0.1_Pd_0.9_/rGO	25	–	105	–	–	[51]

^a^ TOF was calculated after 50 min. ^b^ TOF was calculated after 160 min. ^c^ The ratio between FA and sodium formate was 1:90. ^d^ TOF was calculated based on the surface metal sites.

## Data Availability

Data are contained within the article.
